# The evolution of cooperative breeding in family groups: when should parents tolerate unhelpful helpers?

**DOI:** 10.1098/rstb.2023.0275

**Published:** 2025-03-20

**Authors:** António M. M. Rodrigues, Christina Riehl

**Affiliations:** ^1^ Department of Integrative Biology, University of California, Berkeley, CA 94720, USA; ^2^ Department of Ecology and Conservation Biology, Texas A&M University, College Station, TX 77843, USA; ^3^ Ecology and Evolutionary Biology Program, Texas A&M University, College Station, TX 77843, USA; ^4^ Department of Ecology and Evolutionary Biology, Princeton University, Princeton, NJ 08544, USA

**Keywords:** class structure, constant helper hypothesis, inclusive fitness, kin competition, reproductive scheduling, sibling competition

## Abstract

Cooperatively breeding vertebrates typically live in family groups in which some offspring delay breeding and remain on the natal territory to help rear younger siblings. However, field studies find that helpers can have a neutral or even negative effect on the survival of their relatives. Why, then, do helpers remain, and why do parents tolerate them? Here, we use a kin selection approach to model the conditions under which tolerating helpers is adaptive to parents. Unlike previous models, we consider scenarios in which relatives compete for breeding opportunities in a saturated habitat. We show that kin competition is sufficient to favour tolerance of helpers, even when helpers decrease parental survival or fecundity. Helping is additionally favoured when delaying dispersal benefits the helper (either by decreasing the costs of dispersal or by increasing the chance of territory inheritance). This suggests that the division of reproduction in cooperative family groups can emerge for reasons unrelated to the effects of help itself, but the resulting society sets the stage for more elaborate forms of division of labour. Kin-based helping may therefore be adaptive not only because helpers are related to the brood whom they help, but also because delayed breeding reduces reproductive conflict among siblings.

This article is part of the theme issue ‘Division of labour as a key driver of social evolution’.

## 1. Introduction

Cooperatively breeding vertebrates, including birds, mammals and some fish, typically live in small family groups in which some adult offspring—helpers—delay dispersal and remain on the natal territory to help rear younger relatives. These societies have long been used to explore the fitness trade-offs that influence the evolution of cooperative behaviours, since the reproductive division of labour between breeders and helpers is thought to represent a key transition in the evolution of social complexity [1,2]. Unlike eusocial species, in which non-reproductive group members remain permanently sterile, helpers in cooperative breeding groups retain the ability to reproduce themselves. Delayed reproduction therefore reflects a trade-off between the helper’s current and future reproductive prospects, and between direct and indirect components of their inclusive fitness [3]. Understanding the selective pressures that influence these trade-offs, and hence, the division of labour into reproduction and offspring care, remains a central challenge in the study of both cooperative breeding and eusociality.

Studies of cooperatively breeding birds provided some of the earliest empirical tests of Hamilton’s [4] formulation of kin selection, finding that, under some circumstances, the indirect fitness benefits of rearing younger relatives could partly compensate for the direct fitness costs of delayed reproduction [5,6]. These studies emphasized the indirect fitness benefits of helping, which required a positive effect of helpers on the survival or condition of the brood that they helped [7,8] and on high relatedness between the helper and the brood [9,10]. The subsequent several decades of field studies have largely affirmed the importance of kin selection in the evolution of cooperative breeding (reviewed in [11,12], as well as the importance of ecological constraints on independent breeding that favour delayed dispersal (e.g. shortages of mates or breeding sites [13].

However, evidence that helpers increase the reproductive output of breeders remains puzzlingly inconsistent. In some species, helpers have an obvious positive effect on the survival of the brood [14–16], whereas in others the effect of helpers appears to be neutral [17,18] or even negative [19,20]. Many long-term studies find that the effect of helpers varies in magnitude and even direction across years [21,22]. In acorn woodpeckers (*Melanerpes formicivorus*), male helpers have positive effects on brood survival only in resource-rich years, suggesting that in times of scarcity, helpers decrease their contributions to the brood or even compete with the brood for limited food on the territory [23]. Similarly, removal experiments in Seychelles warblers (*Acrocephalus seychellensis*) demonstrated that while the presence of one helper increased the reproductive success of its parents, more than one helper had the opposite effect, apparently due to competition between helpers and the brood (and/or the breeders [24]. A recent meta-analysis of studies that controlled for potential confounds (for example, territory quality and the prior reproductive success of the pair) found that helpers significantly increased breeder fitness in only 7 of 19 species, although the direction of the effect was positive in all but three species [25].

Several non-mutually exclusive hypotheses have been proposed to explain the adaptive benefits of cooperative breeding in these societies, despite the apparent unhelpfulness of helpers. One well-supported possibility is that helpers increase breeder fitness by allowing breeders to reduce their own workload and therefore divert those resources to self-maintenance or future reproduction (‘’ [26–29]). Second, helping may be favoured by direct fitness benefits that accrue to the helper, such as increased survival or likelihood of inheriting a breeding territory, relative to an individual who disperses [13,30,31]. If the direct fitness benefits of helping are sufficiently high (or, equivalently, the prospects of dispersing to breed are sufficiently low), then helpers could benefit from remaining on the natal territory even if their effects on the fitness of the brood are neutral or negative. Allowing a helper to delay dispersal and remain at home may therefore be viewed as a form of extended parental care by the breeder towards the helper [32,33]. Tolerance of helpers might additionally increase the parent’s inclusive fitness if it reduces competition among siblings over breeding opportunities that arise on neighbouring territories, although this possibility has not been explicitly modelled [34–36]. From the inclusive-fitness perspective of the parent, the key questions are (i) to what extent does delayed dispersal increase the lifetime reproductive output of the helper, and (ii) does this positive fitness effect compensate for potentially negative effects on the brood that is helped?

In this article, we present a model to explore the conditions under which parents should tolerate helpers on the natal territory. We assume a situation in which the breeding habitat is fully saturated, territories become available only when parents die, and offspring from broods compete with one another for these breeding territories (through either territory inheritance or dispersal into neighbouring sites). We explore scenarios in which delayed dispersal has positive effects on helper fitness (for example, by increasing their body condition and therefore reducing the eventual costs of dispersal to a breeding site), and/or negative effects on the survival or reproductive output of breeders; and we compare these to a baseline scenario in which helping affects neither the dispersal costs to helpers nor the survival or fecundity of breeders.

## 2. Methods

### (a) Life cycle

**Figure 1.**
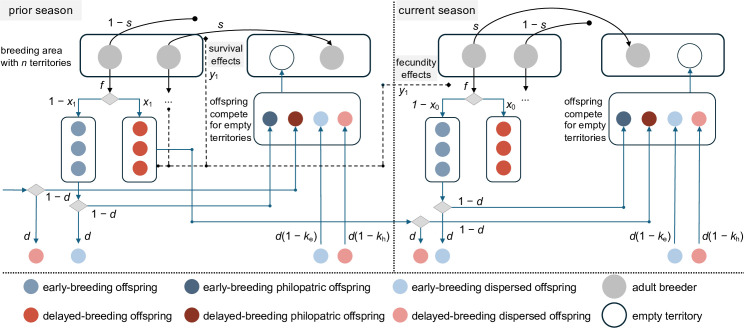
Conceptual schematic representation of the model’s life cycle. We depict two seasons, each comprising a reproductive and a survival stage. Offspring face two key decisions over the course of their lifespan: (i) to become a helper (*x*) or an early breeder (1 – *x*); and (ii) to attempt breeding locally (1 – *d*) or elsewhere (*d*). Early breeders experience equal or higher costs of dispersal relative to helpers (*k*_e_≥ *k*_h_). During the survival stage, maternal mortality may occur, leading to available territories for offspring. The four types of offspring compete for those territories. Production of helpers (illustrated by the average phenotype *y*) may influence the survival and/or fecundity of breeders.

We assume a population divided into breeding groups, with each group comprising *n* asexually reproducing haploid breeding females, each occupying its own breeding territory. Within these breeding groups, mothers produce broods containing *f*(**z**) offspring, where **z** is a vector representing the phenotype of mothers within the group, as elaborated below. After reproducing, mothers survive with probability *s*(*z*), where *z* is the mean phenotype among the local mothers in the current breeding season. Offspring develop into 1-year-olds, with a fraction 1 – *z* of these young adults attempting breeding in the current season and a fraction *z* delaying their breeding attempt by one season and acting as helpers. For a focal mother in the local breeding area, *z* denotes the ratio of offspring who delay breeding (helpers) to those who attempt to breed. Among the 1 – *z* fraction of offspring attempting breeding in the current season, they can either attempt breeding in one of the *n* local territories, with probability 1 – *d*, or disperse and attempt breeding elsewhere, with probability *d*. The probability that these individuals successfully reach a new breeding area is determined by 1 – *k*_e_, where *k*_e_ represents the cost of dispersal for offspring who attempt to breed in their first season as adults. As previously described, a fraction *z* of the *f*(**z**) offspring born in the current season delay breeding by one season, remaining in the natal territory as helpers. They then attempt breeding in their second season as adults. Like their early-breeding siblings, they can either attempt breeding locally, with probability 1 – *d*, or opt to disperse, with probability *d*. However, we assume that delayed breeding reduces the eventual costs of dispersal when they do disperse, either because their body condition improves during the time spent on the natal territory, or for other reasons (e.g. the ability to survive dispersal may increase with age or experience). Specifically, we consider that delayed-breeders (helpers) survive dispersal with probability 1 – *k*_h_, where *k*_h_ represents their cost of dispersal. This cost may be equal to or lower than the cost of dispersal for early-breeders, i.e. *k*_h _≤ *k*_e_. Following the dispersal stage, all offspring, including those born in the current and previous seasons, compete for available breeding territories in different areas. These breeding territories become available when resident mothers die, which occurs with probability 1 – *s*(*z*). At this stage, the life cycle resumes. A schematic depiction of the life cycle is presented in figure 1 and parameters are summarized in table 1.

**Table 1.** Summary of key model parameters.

symbol	meaning
*c*_s_ (*c*_f_)	maternal mortality (fecundity) cost owing to the presence of helpers
*d*	long-distance dispersal
*f*	brood size
*k*_e_ (*k*_h_)	cost of long-distance dispersal for early-breeding offspring (helpers)
*n*	number of breeding females and territories in the focal patch
*s*	survival rate of breeding females
*ω*_e_	fitness of early-breeding offspring
*ω*_h_	fitness of delayed-breeding offspring (i.e. helpers)

### (b) Analysis

To analyse the model, we employ the neighbour-modulated approach to kin selection within a class-structure framework [37–39]. Our objective is to identify the optimal brood composition, denoted as *z* ^*^, from the inclusive-fitness perspective of the mother. This represents the strategy where the selection gradient is null, at which point there is no selection for higher or lower values of *z* ^*^. In the first step, we apply the neighbour-modulated approach to find the selection gradient, which we then interpret in terms of the inclusive-fitness effect of the behaviour [38,40,41]. Below, we summarize the steps necessary to derive the selection gradient. A comprehensive explanation of the methods is provided in the electronic supplementary material, appendices A–G. Within the class-structure framework, individuals in a breeding area are categorized into two classes: breeders and helpers (offspring that delay breeding). Total fitness is described by *w*(**x**,**y**) = **vA**(**x**,**y**)**u**, where **v** is a row vector containing the reproductive values of breeders and helpers; **u** is a column vector containing the stable class-frequencies of the two classes and **A**(**x**,**y**) is the full (gametic) fitness matrix, where **x** is a vector representing the phenotypes of focal recipients and **y** is a vector representing the phenotype of a set of actors within the focal group (explained in more detail below). The vector **v** is determined by the dominant left-eigenvector of the normal fitness matrix **A** ^*^, while the vector **u** is determined by the dominant right-eigenvector of the normal fitness matrix **A** ^*^, where the normal fitness matrix assumes a neutral population, where **x** = **y** = **z** and **A** ^*^ = **A**(**z**,**z**), with **z** representing the population’s average phenotypic values. The full fitness matrix can be written as:


(2.1) $$A\left(x,y\right)=\underset{A^{\phi }\left(x,y\right)}{\underbrace{\left(\begin{array}{cc} w_{h\to h}^{\phi }\left(x,y\right) & w_{b\to h}^{\phi }\left(x,y\right)\\ w_{h\to b}^{\phi }\left(x,y\right) & w_{b\to b}^{\phi }\left(x,y\right) \end{array}\right)}}+\underset{A^{\delta }\left(x,\mathrm{y,}\right)}{\underbrace{\left(\begin{array}{cc} w_{h\to h}^{\delta }\left(x,y\right) & w_{b\to h}^{\delta }\left(x,y\right)\\ w_{h\to b}^{\delta }\left(x,y\right) & w_{b\to b}^{\delta }\left(x,y\right) \end{array}\right)}}$$

where **A** *^ϕ^* (**x**,**y**) is the ‘philopatric’ fitness matrix containing all the fitness components (including adult survival and production of offspring) generated through individuals that remain in the local patch, and **A** *^δ^* (**x**,**y**) is the ‘dispersed-offspring’ fitness matrix containing all the fitness components generated through dispersed offspring [41]. In both fitness matrices, the columns describe the recipients of each class. The first column represents a focal helper (subscript ‘h’) and the second column represents a focal breeder (subscript ‘b’). On the other hand, the rows of the fitness matrices represent the genetic contributions of each recipient to the different classes. The first row corresponds to contributions to the class of helpers, and the second row corresponds to contributions to the class of breeders.

We use ℊ to denote the breeding value at the locus responsible for the trait of interest. The selection gradient is then given by the derivative of total fitness with respect to the breeding value:


(2.2) $$\frac{dw\left(x,y\right)}{dg}=v\left(\frac{\partial A\left(x,y\right)}{\partial x_{0}}+\frac{\partial A\left(x,y\right)}{\partial y_{0}}\circ R_{0}+\frac{\partial A\left(x,y\right)}{\partial y_{1}}\circ R_{1}+\frac{\partial A\left(x,y\right)}{\partial y_{2}}\circ R_{2}\right)u,$$

where *x*_0_ represents the phenotype of the current-season focal mother; *y*_0_ represents the ‘whole-class’ average phenotype among current-season mothers; *y*_1_ represents the average phenotype among prior-season mothers and *y*_2_ is the average phenotype among two-prior-season mothers (see figure 1 for a schematic representation of the phenotypes). The operator ‘∘’ describes the entrywise binary operation between two matrices [41]. The matrices **R** share the dimensions of **A**, and they include the different coefficients of relatedness between actors and recipients. The matrix **R**_0_ contains the coefficients of relatedness between the current-season breeders and a focal current-season breeder (first column), as well as a focal helper (second column). The matrix **R**_1_ contains the coefficients of relatedness between the prior-season breeders and a focal helper (first column) and a focal helper (second column). The matrix **R**_2_ contains the coefficients of relatedness between the two-prior-season breeders and a focal current-season breeder (first column) and a focal helper (second column).

We express the selection gradient in terms of the inclusive-fitness effect of the behaviour [37,38,41]. Specifically, we find that the inclusive-fitness effect is given by


(2.3) $$w_{IF}=w_{IF}^{h}+w_{IF}^{s}+w_{IF}^{f},$$

where $w_{IF}^{h}$ describes the inclusive-fitness effect of an additional number of helpers when helpers do not impact the survival or fecundity of breeders; $w_{IF}^{s}$ describes the inclusive-fitness effects arising from the impact of helpers on the survival of breeders and $w_{IF}^{f}$ describes the effects arising from the impact of helpers on the fecundity of breeders. In the absence of survival and fecundity effects, the inclusive-fitness effect of producing additional helpers is given by


(2.4) $$w_{IF}^{h}=f\left(-\omega_{e}+\omega_{h}\right)+f\varphi^{2}\left(\left(1-z\right)R_{\omega }+zr_{\beta }\right)-f\varphi^{2}\left(\left(1-z\right)r_{1}+zR_{\omega }\right),$$

The first term describes the direct fitness effect when a mother produces more helpers instead of early-breeding offspring, where *ω*_e_ represents the success of an early-breeding offspring and *ω*_h_ the success of helpers (delayed-breeding offspring). The second and third terms represent the reduction in kin competition in the current season and the corresponding increase in the next season owing to the focal mother’s additional production of helpers, who delay breeding for one season. Specifically, the focal mother produces fewer offspring competing in the current season, who would have acquired local resources with probability *φ*, but produces more offspring competing in the next season, who acquire resources with probability *φ*. This frees up resources in the current season, which are acquired by philopatric offspring with probability *φ*. With a probability 1 – *z*, these philopatric offspring are early-breeding offspring, related to the actor by *R*_ω_. Conversely, with probability *z*, they are delayed-breeding offspring, related to the actor by *r*_β_. The additional helpers displace philopatric offspring competing in the next season, who would have otherwise obtained breeding territories with a probability *φ*. With probability 1 – *z*, the displaced philopatric offspring are early-breeding offspring born in the next season, related to the focal mother by *r*_1_. With probability *z*, the displaced offspring are delayed-breeding offspring (helpers) born in the current season, related to the focal mother by *R*_ω_.

When helpers at the nest influence the survival of mothers, the inclusive-fitness effect resulting from the increased production of helpers through their effect on survival is given by:


(2.5) $$w_{IF}^{s}=\frac{f}{1-s}c_{s}\left(-R_{\omega }+\varphi \left(\left(1-z\right)R_{\omega }+zr_{\beta }\right)\right)$$

When a mother produces more helpers, she increases the mortality of local females (including herself) by *c*_s_, whose relatedness to the focal mother is *R*_ω_. These affected mothers would have otherwise generated a reproductive value *f*/(1 – *s*). Simultaneously, increased maternal mortality releases breeding territories that are subsequently acquired by philopatric offspring with a probability *φ*. With probability 1 – *z*, these offspring are early-breeding offspring whose relatedness to the actor is *R*_ω_, while with probability *z*, they are delayed-breeding offspring whose relatedness to the actor is *r*_β_. Once offspring secure territories and transition into breeders, their reproductive value becomes *f*/(1 – *s*).

When helpers at the nest affect the fecundity of breeders, there are three inclusive-fitness effects to consider:


(2.6) $$w_{IF}^{f}=c_{f}\left(-\omega_{o}r_{1}+\varphi^{2}\left(\left(1-z\right)\left(\left(1-z\right)r_{\beta }+zR_{\omega }\right)+z\left(\left(1-z\right)r_{2}+zr_{1}\right)\right)\right)$$

First, when the focal mother produces more helpers, next-season females produce *c*_f_ fewer offspring, who would have contributed *ω*_o_ to fitness. This fitness cost must be depreciated by the relatedness between the mother and next-season breeders, denoted as *r*_1_. The production of fewer offspring by next-season females reduces the intensity of kin competition in subsequent seasons. With a probability of *φ*, these offspring would have secured breeding resources that now become available for other offspring, either in the next season, with probability 1 – *z*, or the season after, with probability *z*. With a probability of 1 – *z*, the resources that become available in the next season are acquired by the philopatric early-breeding offspring produced in the next season, whose relatedness to the actor is *r*_β_, or, with probability *z*, by philopatric delayed-breeding offspring produced in the current season, whose relatedness to the actor is *R*_ω_. The resources that become available the season after next are acquired by philopatric early-breeding offspring produced two seasons into the future, with probability 1 – *z*, whose relatedness to the actor is *r*_1_, or by philopatric delayed-breeding offspring born one season into the future, with probability *z*, whose relatedness to the actor is *r*_2_.

## 3. Results

### (a) An invariance result: selection for helpers that do not help

In this scenario, we assume that delaying reproduction does not reduce the costs of dispersal. Consequently, both early- and delayed-breeding offspring incur the same dispersal cost, denoted as *k*_e_ = *k*_h_ = *k*. Additionally, we assume that the presence of delayed-breeding individuals (helpers) at the nest has no effect on the survival of mothers (i.e. *s*(*z*) = *s*) or their fecundity (i.e. *f*(**z**) = *f*).

**Figure 2.**
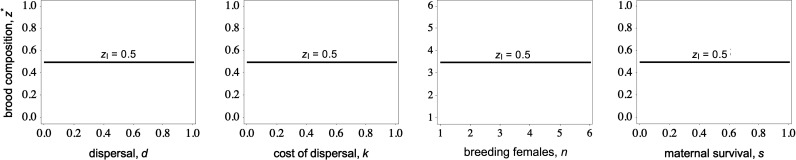
Optimal brood composition as a function of different traits and ecological variables when delaying breeding does not impact dispersal costs, i.e. *k*_e_ = *k*_h_. Mothers are favoured to produce broods with precisely half early- and half delayed-breeding offspring (*z*_I_ = 0.5 or 50 : 50 broods) irrespective of the dispersal rate, *d*, the cost of dispersal, *k*, group size, *n*, and maternal survival, *s*. This invariance result also holds for variation in the fecundity rate, *f*.

Our findings show that mothers are selected to produce broods with precisely half delaying-breeding offspring (helpers) and half early-breeding offspring (figure 2, Box 1). Moreover, our model demonstrates that this ‘offspring-ratio’ strategy remains independent of multiple ecological and demographic factors. These factors include the fecundity (*f *) and survival (*s*) of mothers, the number of mothers in the breeding group (*n*), the degree of dispersal (*d*) and the associated cost of dispersal (*k*). We refer to this invariance as the ‘constant helper hypothesis’ (CHH), drawing parallels with the ‘constant male hypothesis’ of the sex allocation theory [38,42–44], and the ‘constant philopater hypothesis’ of the dispersal evolution theory [45].

Box 1.
**An invariance result for cooperative breeding.**

Here, we consider a baseline scenario where delaying breeding does not affect offspring body condition (*k*_e_ = *k*_h_) and we focus on the inclusive-fitness implications of producing helpers, as described by equation (2.4) in the main text. In this scenario, the focal mother produces more delayed-breeding offspring while producing fewer early-breeding offspring. Since delaying breeding does not impact offspring body condition (*k*_e_ = *k*_h_), both kinds of offspring exhibit identical fitness (*ω*_e_ = *ω*_h_), resulting in a neutral net effect on the mother’s direct fitness. However, the production of helpers impacts the indirect fitness component that describes the level of kin competition experienced by the different offspring. Under this scenario, the inclusive-fitness effect becomes

(3.1) .$$w_{IF,I}=\varphi^{2}\left(\left(\left(1-z\right)R_{\omega }+zr_{\beta }\right)-\left(\left(1-z\right)r_{1}+zR_{\omega }\right)\right)$$

Note that the relatedness between the focal actor and the delayed-breeding offspring born in the previous season (*r*_β_) is identical to that between the focal actor and the early-breeding offspring born in the next season (*r*_1_). Hence, *r*_1_ = *r*_β_ (electronic supplementary material, appendix E). Thus, the inclusive-fitness effect becomes

(3.2) $$w_{IF,I}=\varphi^{2}\left(\left(1-z\right)\left(R_{\omega }-r_{\beta }\right)-z\left(R_{\omega }-r_{\beta }\right)\right)=\varphi^{2}\left(1-2z\right)\left(R_{\omega }-r_{\beta }\right).$$

The optimal strategy leading to a null selection gradient is therefore *z* ^*^ = ½. Note that this strategy remains invariant across most of the model parameters. Specifically, mothers are selected to produce broods comprising half early-breeding offspring and half delayed-breeding offspring regardless of the number of breeding territories in the local patch (*n*), offspring dispersal rate (*d*), maternal survival (*s*), fecundity of breeders (*f *) and the cost of dispersal (*k*). This invariance result has two significant implications. First, it highlights that selection for ‘helpers’ at the nest exists even in the absence of any actual help. Second, it establishes a baseline to aid in analysing and explaining variation in the number of helpers at the nest. For instance, as illustrated in §3, our model predicts the presence of helpers at the nest even when they negatively impact maternal survival. Observing helpers at the nest could prompt two potential explanations: first, an (erroneous) assumption that helpers do not adversely impact the survival of mothers; and second, a search for benefits for mothers or offspring justifying the presence of these helpers at the nest. However, by basing our analysis on deviations from the baseline scenario, it becomes clear that there are indeed costs for mothers (or offspring) and an absence of benefits (either for mother or offspring), as mothers are producing fewer helpers than those predicted by the baseline scenario.

Why are mothers favoured to produce broods with equal proportions of delaying- and early-breeding offspring? First, when delaying breeding does not affect dispersal costs (*k*_e_ = *k*_h_), the lifetime direct fitness of delayed- and early-breeding offspring are exactly the same (*ω*_e_ = *ω*_h_ in equation (2.4)). This baseline model assumes that delayed breeders and early breeders are equally likely to survive to reproduce. This includes scenarios where helpers experience some mortality during their additional ‘waiting season’ as long as the lifetime direct fitness of delayed- and early-breeding offspring remains equal (*ω*_e_ = *ω*_h_). Specifically, if the survival of helpers during the ‘waiting season’ is denoted by *s*_w_ and their survival outside the ‘waiting season’ by *s*_o,h_, then, as long as *s*_o,e_ = *s*_w_ *s*_o,h_ (where *s*_o,e_ is the survival of early-breeding offspring), we have *ω*_e_ = *ω*_h_, and the baseline is recovered. In cases where offspring delay breeding because their natal patch provides a ‘safe haven’ with little mortality or no mortality, *s*_w_ = 1, and as long as *s*_o,e_ = *s*_o,h_, we gain recover the baseline model with *ω*_e_ = *ω*_h_. More generally, this is true when *s*_o,e_ = *s*_w_ *s*_o,h_, *s*_w_ *s*_o,h_/*s*_o,e_ = 1, regardless of the value of *s*_w_, providing the general condition for our baseline model.

However, we find that the production of delayed-breeding offspring influences the indirect component of fitness (second and third terms in equation (2.4)). Specifically, it reduces the intensity of competition for philopatric early-breeding offspring. This decoupling of competition among related offspring generates indirect fitness benefits for mothers that are partially offset by the costs of additional competition imposed on offspring attempting to breed in the next season. Because the average relatedness between the focal mother and the offspring attempting to breed in the current season and between the focal mother and those attempting to breed in the next season is exactly the same, the equilibrium offspring-ratio strategy depends on the balance between the costs and benefits of decoupling kin competition, rather than relatedness.

We find that the benefit of delayed-breeding offspring is proportional to the fraction of philopatric offspring attempting to breed in the current season, given by 1 – *z*, while the cost is proportional to the fraction of philopatric offspring delaying breeding, given by *z*. Thus, the balance of these benefits and costs occurs when mothers produce broods comprising half early-breeding offspring and half delayed-breeding offspring (i.e. *z*_I_ = ½), which is independent of ecology and demography. This invariance result provides a fundamental baseline for the subsequent results presented below.

### (b) Dispersal benefits favour higher production of helpers

**Figure 3.**
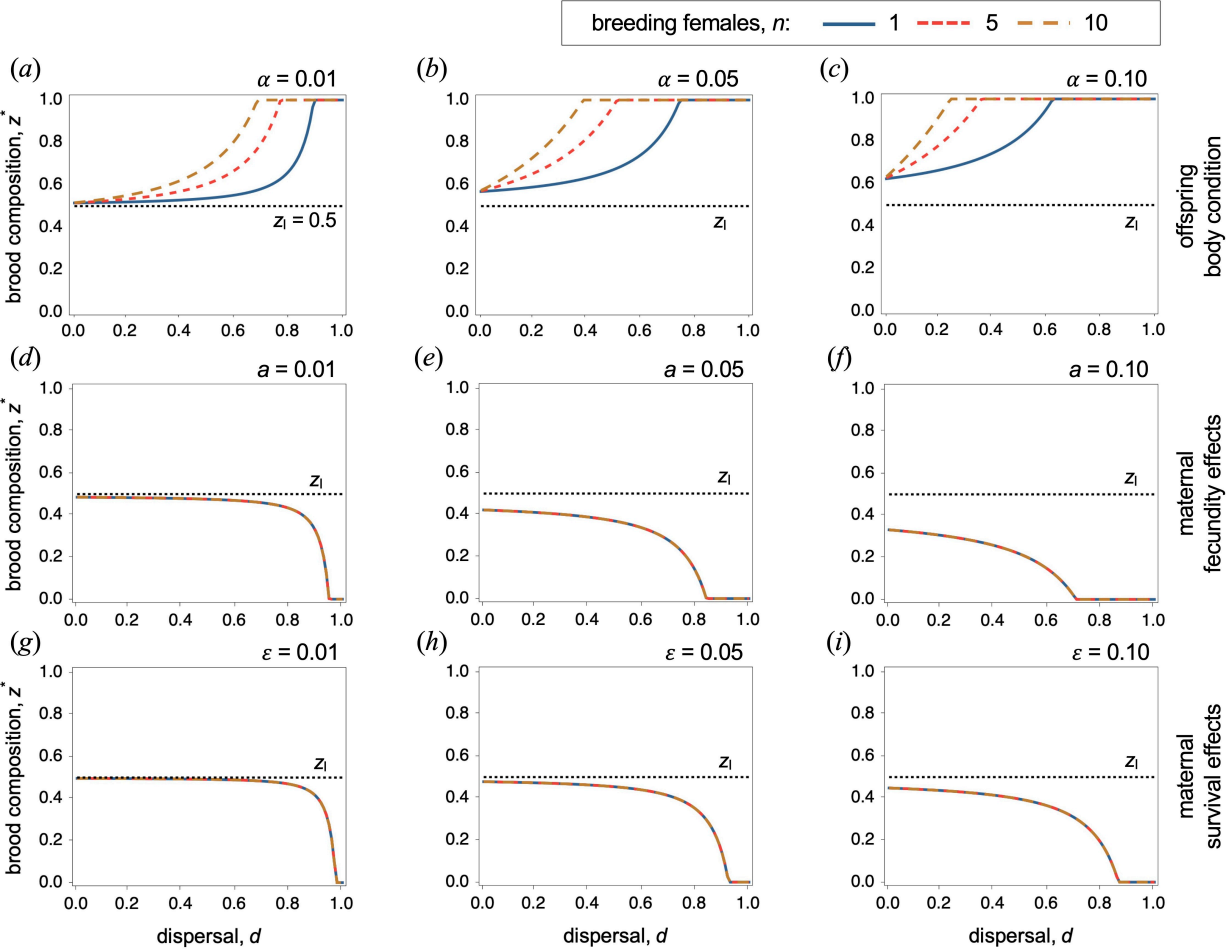
Optimal brood composition as a function of dispersal for different numbers of breeding females in a given area (i.e. local density of breeding females, *n*) assuming that: (*a–c*) delayed breeding improves offspring body condition *α* (with *k*_h_ = *k*_e_ – *α*); (*d–f*) helpers reduce the fecundity of breeders; (*g–i*) and helpers increase maternal mortality. (*a–c*) Mothers are selected to increase the production of delayed-breeding offspring compared with the 50 : 50 brood composition baseline scenario (*z*_I_). A larger number of breeders per breeding area (high *n*) promotes investment in delayed-breeding offspring. (*d–i*) When dispersal is low, mothers are favoured to produce broods close to the 50 : 50 baseline scenario. By contrast, when dispersal is high, mothers are favoured to produce a larger majority of early-breeding offspring. When baseline survival is lower, mothers tend to produce broods closer to the 50 : 50 baseline scenario. Optimal brood composition remains independent of group size. Parameter values: (*a–i*) *s*_0_ = 0.5; (*a–c*) *k*_e_ = 0.5; (*d–f*) *k*_e_ = *k*_h_ = 0.5; (*g–i*) *k*_e_ = *k*_h_ = 0.5, *σ* = 0.01.

In this scenario, we assume that delaying reproduction improves the likelihood that offspring will eventually survive dispersal, resulting in *k*_h_< *k*_e_. Our findings reveal a departure from the 50 : 50 brood invariance result when delayed breeding reduces the eventual costs of dispersal (for example, when helpers are able to improve their body condition by remaining on the natal territory for an additional year, relative to offspring who disperse in their first year). In this context, mothers are now favoured to produce more than half delayed-breeding offspring (figure 3*a–c*). This deviation from the 50 : 50 strategy is directly proportional to the reduction in dispersal costs. When the benefits are marginal, the deviation is modest; however, in cases of substantial benefits, mothers may even be favoured to produce broods exclusively composed of delayed-breeding offspring.

The excess of delayed-breeding offspring is driven by direct fitness effects. As discussed above, when delayed breeding has no impact on dispersal, the fitness of early- and delayed-breeding offspring is the same. However, if delaying breeding results in reduced dispersal costs, delayed-breeding offspring exhibit enhanced fitness, prompting mothers to produce an excess of delayed-breeding offspring, where ‘excess’ means a positive deviation from the 50 : 50 brood composition invariance result established above (*z* ^*^> *z*_I_).

### (c) Mothers produce fewer helpers when helpers reduce breeder fecundity

We now consider a scenario where the presence of offspring (helpers) at the nest imposes a cost on the fecundity of breeders (i.e. helpers reduce the survival of the brood that they help). In this context, when mothers adjust their brood composition by producing more helpers, the fecundity of mothers within the breeding group decreases, with *f*_0_(*y*_1_) = *f*_o_(1 – *ay*_1_) and *c*_f_(*z*) = ∂*f*_0_(*y*_1_)/∂*y*_1_|*_y_*_₁_ *_= z_*, where *f*_o_ is the baseline fecundity, *a* ( ≪ 1) a scaling factor, and *y*_1_ is the local average investment in helpers among prior-season breeding females.

When helpers reduce brood survival, mothers are favoured to produce fewer helpers, with the proportion of these offspring increasing as dispersal decreases (figure 3*d–f*). As noted earlier, reduced fecundity has both a direct negative impact on the reproductive output of breeders of breeders and a positive effect by decreasing kin competition. However, in cases of full dispersal, the kin competition benefit vanishes, leaving only the negative impact of helpers at the nest. Consequently, when dispersal rates are higher, mothers tend to reduce investment in delayed-breeding offspring. Conversely, when dispersal rates are extremely low, the benefits of reduced kin competition can be sufficient to offset the fecundity costs associated with the presence of helpers. As a result, mothers tend to produce broods that are closer to the 50 : 50 brood invariance.

Maternal survival significantly influences the extent to which mothers produce delayed-breeding offspring. When survival rates are low, the relatedness between breeders in consecutive seasons decreases. This consequently reduces the fecundity costs to mothers arising from the production of helpers. Thus, in situations with lower maternal survival rates, mothers tend to produce broods that are closer to the 50 : 50 broods invariance result. Additionally, we observe another important invariance in the strategy of mothers: the optimal brood composition of mothers is independent of the number of breeders residing in each patch (figure 3*d–f*).

### (d) Production of helpers is favoured even when helpers reduce maternal survival

So far, we have explored cases in which the presence of offspring (helpers) at the nest imposes no costs on the survival of breeders. Here, we consider cases where helpers influence the survival of breeding females. To study these cases, we revisit our baseline model, introducing a crucial modification. Specifically, we consider that the presence of delayed-breeding offspring (helpers) at the nest has a negative impact on the survival of breeders, with *s*(*y*_0_) = *s*_o_ + *σ*(1 – *y*_0_)*^ε^*, and *c*_s_(*z*) = ∂*s*(*y*_0_)/∂*y*_0_|*_y_*_₀ =_ *_z_*, where *s*_0_ is the baseline survival, and *σ* and *ε* are scaling factors.

Our findings show that the optimal offspring-ratio strategy of mothers strongly depends on the dispersal rate (figure 3*g–i*). When the dispersal of offspring is relatively low, the effects of maternal mortality on the optimal brood composition are marginal. Mothers tend to produce broods with approximately half delayed- and half early-breeding offspring, mirroring the baseline model. This weak influence of mortality effects on the invariance result stems from two distinct fitness consequences from mortality effects. On the one hand, the production of delayed-breeding offspring negatively impacts the survival of mothers, contributing negatively to their direct fitness. On the other hand, the increased mortality of mothers has a secondary effect impacting the indirect component of a mother’s fitness. As mothers die, they release resources, and when offspring display a tendency to remain philopatric, these resources are likely to be acquired by their own or closely related offspring. These two direct and indirect effects acting in opposite directions nearly cancel each other out, such that survival effects result in minimal deviations from the established invariance result (*z* ^*^≈ *z*_I_).

In contrast, under conditions of relatively high dispersal, these results change dramatically and survival effects can substantially influence the invariance result. This shift occurs because the second indirect effect of increased maternal mortality nearly vanishes when dispersal is high. The first direct effect persists: the presence of helpers at the nest has a negative impact on the direct fitness of local mothers. However, the second indirect effect is notably reduced: a high dispersal rate means that the resources released by mothers upon their demise are likely to be acquired by dispersed unrelated offspring, rather than philopatric-related offspring. Consequently, the secondary positive indirect fitness effect of increased maternal mortality vanishes, and the negative direct fitness effects become the dominant factor in the selection gradient. This prompts mothers to reduce the proportion of offspring that delay breeding and remain at the nest (*z* ^*^< *z*_I_).

**Figure 4.**
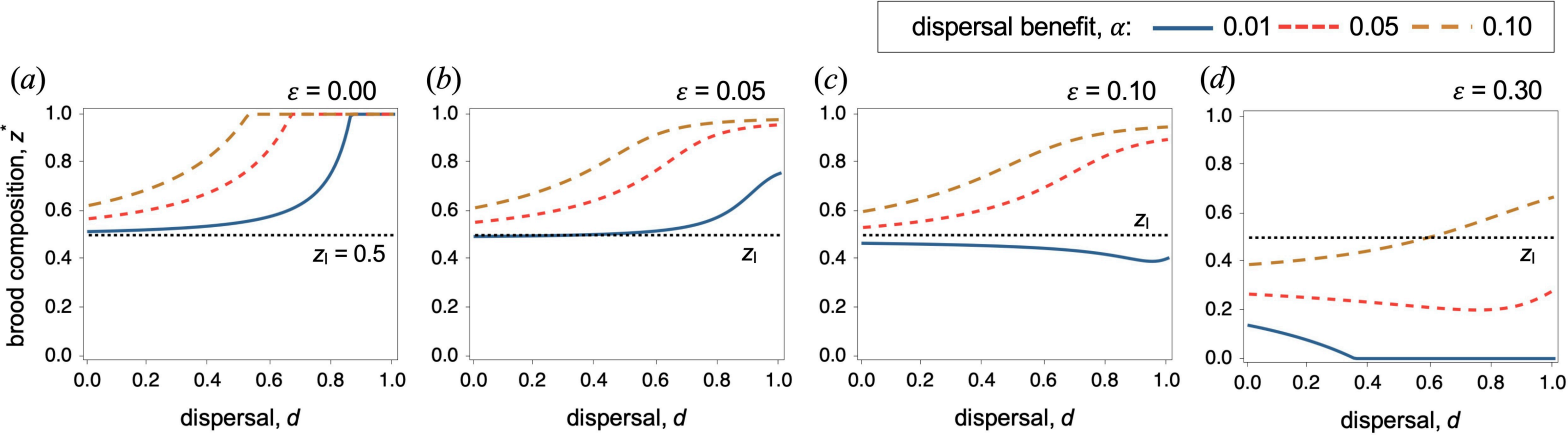
Optimal brood composition as a function of dispersal and the benefit of delaying breeding, *α* (with *k*_h_ = *k*_e_ – *α*), for varying maternal survival costs owing to the presence of helpers. In the absence of benefits (*α* = 0, and *k*_h_ = *k*_e_), mothers produce 50 : 50 broods. However, as the benefit of delaying breeding (*α*) and the rate of dispersal (*d*) increase, mothers are selected to gradually produce more delayed-breeding offspring. If helpers reduce maternal survival, this offsets some of the benefits of delaying offspring and mothers may produce broods in which less than half of their offspring become helpers. Parameter values: *k*_e_ = 0.5, *n* = 2, *s*_0_ = 0.5, *σ* = 0.01.

Our findings also reveal an invariance that still holds when survival effects are considered. Specifically, we find that the optimal composition of broods is independent of the number of breeders in each patch (figure 3*g–i*). More generally, delaying breeding may be simultaneously beneficial (e.g. lower cost of dispersal) for offspring but detrimental for mothers (e.g. increased mortality). In such cases, mothers may produce more or fewer helpers depending on the magnitude of these two opposing selective pressures (figure 4).

### (e) Co-evolution between breeding strategy and dispersal

As we have seen above, when delaying breeding affects the ability of offspring to survive dispersal, their tendency to delay breeding depends on the level of dispersal in the population, which we have treated as a fixed model parameter. In natural populations, by contrast, dispersal will likely be an evolving trait and therefore, optimal offspring breeding strategies may depend on how they co-evolve with dispersal. In this section, we explore the consequences of letting dispersal co-evolve with the breeding strategy of offspring (electronic supplementary material, appendix F).

We find two primary forces driving the evolution of dispersal. First, dispersal is favoured when the body condition of helpers increases (electronic supplementary material, figure F1). This is because improved body condition reduces the cost of dispersal, thereby reducing the direct fitness costs of dispersal. In addition, indirect fitness effects also drive the evolution of dispersal. In particular, dispersal improves the fitness of kin that remain in the local group, both early- and delayed-breeding offspring. When the number of breeders is relatively small, this leads to higher relatedness among offspring, which favours the evolution of dispersal. In such cases, we find intermediate levels of optimal dispersal, favouring the evolution of delayed breeding.

### (f) Territory inheritance promotes the evolution of helping

We have assumed that one major advantage of delayed dispersal is that it reduces the eventual costs of dispersal when helpers do leave the natal territory. In many cooperatively breeding species, though, delayed dispersal may also increase the likelihood that a helper inherits a breeding position on the natal territory when the parent(s) die. We next extended the model to include this ‘home-field advantage’ for philopatric offspring by considering a scenario in which, if a vacancy opens, the helper is more likely to inherit it than a newcomer [41]. We find that incorporating this additional advantage leads to increased selection for delayed breeding, compared with the baseline scenario (electronic supplementary material, appendix G). In particular, we find that the production of delayed-breeding offspring increases with the level of dispersal in the population (electronic supplementary material, figure G1). Thus, this mirrors the patterns obtained for the dispersal advantage scenario in §3. However, there are some differences. At lower levels of dispersal, there is a stronger selection for delayed-breeding strategies under the home-field advantage scenario than under the dispersal advantage scenario. By contrast, at higher levels of dispersal, there is a stronger selection for delayed-breeding strategies under the dispersal advantage scenario than under the home-field advantage scenario. These contrasting patterns occur because of the nature of the benefits. Under the dispersal advantage scenario, the magnitude of the advantage increases with dispersal. By contrast, under the home-field advantage scenario, the magnitude of the advantage decreases with dispersal.

## 4. Discussion

Although ecological constraints on independent breeding, particularly competition for breeding vacancies in a saturated habitat, have long been proposed as a major driver of cooperative breeding, the costs of kin competition among siblings across breeding seasons for these vacancies have not been included in quantitative models (e.g. [34–36]). By explicitly including these costs, we find that, in theory, cooperative breeding can be favoured even when the helper gains neither direct fitness through increased survival, nor indirect fitness through helping the brood or their parents. More specifically, mothers are consistently selected to produce a fixed number of helpers regardless of various ecological and demographic parameters, an invariance that we term the ‘constant helper hypothesis’. From this viewpoint, the presence of helpers does not appear to require a special explanation; rather, it emerges naturally from the local demographic dynamics of groups and competition for territories. These results are useful as a null model, highlighting the potential significance of kin competition among siblings (both within and between broods) over breeding opportunities across breeding seasons, which has previously received little theoretical attention (in contrast to, for example, competition among family members for resources on the breeding territory [46–48]). Recent studies have identified other evolutionary explanations for the tolerance of apparently ‘unhelpful’ helpers, which may act in concert with the effects of kin competition that we explore here. In stochastic environments, for example, the presence of helpers may reduce variance in reproductive success over time (bet-hedging), which, under some conditions, can increase inclusive fitness despite reductions in average reproductive output [49]. Although our model assumes stable conditions, environmental fluctuations may play an important role in favouring helping behaviour in natural systems.

More generally, kin competition drives a range of other behavioural strategies—namely, dispersal over longer distances away from the natal territory—that have similarly evolved to minimize kin competition over breeding as well as other negative consequences of local kin structuring (i.e. inbreeding [50]). Crucially, our model shows that the proportion of offspring that delay breeding and become helpers remains independent of the level of long-distance dispersal; hence, it is decoupled from the intensity of kin competition emerging from philopatry. Consequently, our model does not predict a specific correlation between the number of helpers and the level of dispersal. By contrast, in real systems, empirical observations often reveal a lower proportion of helpers compared with dispersers. This contrast between predictions and observations could stem from various factors. For instance, cooperative breeding may be associated with a factor driving high dispersal but not the proportion of helpers. In our model, we find that the number of helpers remains independent of the number of breeders within a group. Theoretical studies have consistently indicated that fewer breeders correlate with increased dispersal rates (e.g. [51]. Therefore, if cooperative breeding involves fewer breeders per group, it could explain the elevated ratio between dispersers and helpers.

Conceptually, these results are consistent with recent theoretical work showing that competition over reproductive opportunities can favour the evolution of a suite of life-history traits that affect the scheduling of breeding across an animal’s lifespan, including the onset and cessation of reproduction [52,53]. In long-lived species that experience reproductive senescence, such as humans and some cetaceans, empirical studies have generally found that the indirect fitness benefits of help by post-reproductive females (‘grandmothering’ benefits) are insufficient to explain the evolution of menopause because they fail to fully compensate for the potential benefits of continued reproduction [54,55]. Models incorporating both the costs of reproductive competition and the benefits of kin-directed help have been notably more successful at explaining the timing and evolutionary stability of reproductive senescence in these societies [56,57].

Finally, our model results emphasize that kin selection is still a crucial driver in the evolution of family-based cooperative breeding (through the inclusive fitness benefits that accrue to parents who tolerate helpers on the natal territory), regardless of the fitness effects of the help itself. This result can help to explain not only field studies of societies in which offspring care provided by helpers has no measurable effect on brood survival, but also family-living societies in which retained offspring do not help at all [58,59]. These societies are of particular interest in understanding the evolutionary origins of cooperative breeding, since phylogenetic comparative analyses support the hypothesis that family living has preceded helping behaviour across avian lineages [60,61]. Of course, when helping behaviour does increase the fitness of the brood—as is undoubtedly true, in many cases—such benefits should only strengthen selection for cooperative breeding. We show that the division of reproduction between breeders and non-breeders—the most common form of division of labour in cooperative vertebrates—can arise without the fitness benefits of labour itself. However, once these groups are established, non-breeders should be under selection to increase the fitness of their relatives by providing help. More elaborate forms of division of labour could subsequently evolve if (for example) breeders and non-breeders vary in the types of tasks that they can most efficiently provide (e.g. [62]) or if breeders actively prevent non-breeders from participating in certain tasks (e.g. [19,63]; reviewed in [64]). Therefore, these results complement other hypotheses for the evolution of family societies and suggest unifying connections between societies in which ‘helpers’ do not help and those in which they do. Inclusive-fitness models that incorporate both the benefits of helping as well as the costs of kin competition over reproductive opportunities may offer an improved understanding of the evolution of delayed dispersal, helping behaviour and the reproductive division of labour in cooperative societies.

## Data Availability

The model specifications are included in the electronic supplementary material [65].

## References

[1] Bourke AFG . 2011 Principles of social evolution. Oxford, UK: Oxford University Press.

[2] Downing PA , Griffin AS , Cornwallis CK . 2020 Group formation and the evolutionary pathway to complex sociality in birds. Nat. Ecol. Evol. **4** , 479–486. (10.1038/s41559-020-1113-x) 32094543

[3] Cant MA . 2012 Cooperative breeding systems. In The evolution of parental care (eds NJ Royle , PT Smiseth , M Kölliker ), pp. 206–2220. Oxford, UK: Oxford University Press. (10.1093/acprof:oso/9780199692576.003.0012)

[4] Hamilton WD . 1964 The genetical evolution of social behaviour. II. J. Theor. Biol. **7** , 17–52. (10.1016/0022-5193(64)90039-6) 5875340

[5] Reyer HU . 1984 Investment and relatedness: a cost/benefit analysis of breeding and helping in the pied kingfisher (Ceryle rudis). Anim. Behav. **32** , 1163–1178. (10.1016/s0003-3472(84)80233-x)

[6] Emlen ST , Wrege PH . 1989 A test of alternate hypotheses for helping behavior in white-fronted bee-eaters of Kenya. Behav. Ecol. Sociobiol. **25** , 303–319. (10.1007/bf00302988)

[7] Brown JL , Brown ER , Brown SD , Dow DD . 1982 Helpers: effects of experimental removal on reproductive success. Science **215** , 421–422. (10.1126/science.215.4531.421) 17814957

[8] Mumme RL . 1992 Do helpers increase reproductive success? Behav. Ecol. Sociobiol. **31** 319-328. (10.1007/bf00177772)

[9] Emlen ST , Vehrencamp SL , Ligon JD , Rowley I . 1983 Cooperative breeding strategies among birds. In Perspectives in ornithology (eds AH Brush , GA Clark, Jr ), pp. 93–134. Cambridge, UK: Cambridge University Press. (10.1017/cbo9780511759994.006)

[10] Komdeur J . 1994 The effect of kinship on helping in the cooperative breeding Seychelles warbler (Acrocephalus sechellensis). Proc. R. Soc. Lond. B **256** , 47–52. (10.1098/rspb.1994.0047)

[11] Hatchwell BJ . 2009 The evolution of cooperative breeding in birds: kinship, dispersal and life history. Phil. Trans. R. Soc. B **364** , 3217–3227. (10.1098/rstb.2009.0109) 19805429 PMC2781872

[12] Green JP , Freckleton RP , Hatchwell BJ . 2016 Variation in helper effort among cooperatively breeding bird species is consistent with Hamilton’s rule. Nat. Commun. **7** , 12663. (10.1038/ncomms12663) 27554604 PMC4999512

[13] Kingma SA . 2017 Direct benefits explain interspecific variation in helping behaviour among cooperatively breeding birds. Nat. Commun. **8** , 1094. (10.1038/s41467-017-01299-5) 29061969 PMC5653647

[14] Reed JM , Walters JR . 1996 Helper effects on variance components of fitness in the cooperatively breeding red-cockaded woodpecker. Auk **113** , 608–616. (10.2307/4088981)

[15] Woxvold IA , Magrath MJL . 2005 Helping enhances multiple components of reproductive success in the cooperatively breeding apostlebird. J. Anim. Ecol. **74** , 1039–1050. (10.1111/j.1365-2656.2005.01001.x)

[16] Preston SAJ , Briskie JV , Hatchwell BJ . Adult helpers increase the recruitment of closely related offspring in the cooperatively breeding rifleman. Behav. Ecol. **27** , arw087. (10.1093/beheco/arw087) PMC5181526 28028377

[17] Leonard ML , Horn AG , Eden SF . 1989 Does juvenile helping enhance breeder reproductive success? Behav. Ecol. Sociobiol. **25** , 357–361. (10.1007/bf00302993)

[18] Magrath RD , Yezerinac SM . 1997 Facultative helping does not influence reproductive success or survival in cooperatively breeding white-browed scrubwrens. J. Anim. Ecol. **66** , 658–670. (10.2307/5919)

[19] Eguchi K , Yamagishi S , Asai S , Nagata H , Hino T . 2002 Helping does not enhance reproductive success of cooperatively breeding rufous vanga in Madagascar. J. Anim. Ecol. **71** , 123–130. (10.1046/j.0021-8790.2001.00585.x)

[20] Paquet M , Doutrelant C , Hatchwell BJ , Spottiswoode CN , Covas R . 2015 Antagonistic effect of helpers on breeding male and female survival in a cooperatively breeding bird. J. Anim. Ecol. **84** , 1354–1362. (10.1111/1365-2656.12377) 25850564 PMC4557059

[21] Blackmore CJ , Heinsohn R . 2007 Reproductive success and helper effects in the cooperatively breeding grey‐crowned babbler. J. Zool. **273** , 326–332. (10.1111/j.1469-7998.2007.00332.x) 21054379

[22] Riehl C , Smart ZF . 2022 Climate fluctuations influence variation in group size in a cooperative bird. Curr. Biol. **32** , 4264–4269.(10.1016/j.cub.2022.07.057) 35998636

[23] Koenig WD , Walters EL , Barve S . 2019 Does helping-at-the-nest help? The case of the acorn woodpecker. Front. Ecol. Evol. **7** , 272. (10.3389/fevo.2019.00272)

[24] Komdeur J . 1994 Experimental evidence for helping and hindering by previous offspring in the cooperative-breeding Seychelles warbler Acrocephalus sechellensis. Behav. Ecol. Sociobiol. **34** , 175–186. (10.1007/s002650050031)

[25] Downing PA , Griffin AS , Cornwallis CK . 2020 The benefits of help in cooperative birds: nonexistent or difficult to detect? Am. Nat. **195** , 1085–1091. (10.1086/708515) 32469661

[26] Crick HQP . 1992 Load‐lightening in cooperatively breeding birds and the cost of reproduction. Ibis **134** , 56–61. (10.1111/j.1474-919X.1992.tb07230.x)

[27] Khan M , Walters J . 2002 Effects of helpers on breeder survival in the red-cockaded woodpecker (Picoides borealis). Behav. Ecol. Sociobiol. **51** , 336–344. (10.1007/s00265-001-0441-3)

[28] Hammers M , Kingma SA , van Boheemen LA , Sparks AM , Burke T , Dugdale HL , Richardson DS , Komdeur J . 2021 Helpers compensate for age-related declines in parental care and offspring survival in a cooperatively breeding bird. Evol. Lett. **5** , 143–153. (10.1002/evl3.213) 33868710 PMC8045936

[29] Downing PA , Griffin AS , Cornwallis CK . 2021 Hard-working helpers contribute to long breeder lifespans in cooperative birds. Phil. Trans. R. Soc. B **376** , 20190742. (10.1098/rstb.2019.0742) 33678023 PMC7938162

[30] Ekman J , Eggers Sö , Griesser M , Tegelström Hå . 2001 Queuing for preferred territories: delayed dispersal of Siberian jays. J. Anim. Ecol. **70** , 317–324. (10.1111/j.1365-2656.2001.00490.x)

[31] Kokko H , Ekman J . 2002 Delayed dispersal as a route to breeding: territorial inheritance, safe havens, and ecological constraints. Am. Nat. **160** , 468–484. (10.1086/342074) 18707523

[32] Ekman J , Griesser M . 2002 Why offspring delay dispersal: experimental evidence for a role of parental tolerance. Proc. R. Soc. Lond. B **269** , 1709–1713. (10.1098/rspb.2002.2082) PMC1691083 12204132

[33] Ekman J , Dickinson JL , Hatchwell BJ , Griesser M . 2004 Delayed dispersal. In Ecology and evolution of cooperative breeding in birds (eds WD Koenig , JL Dickinson ), pp. 35–47. Cambridge, UK: Cambridge University Press. (10.1017/CBO9780511606816.003)

[34] Pen I , Weissing FJ . 2000 Towards a unified theory of cooperative breeding: the role of ecology and life history re-examined. Proc. R. Soc. Lond. B **267** , 2411–2418. (10.1098/rspb.2000.1299) PMC1690831 11133031

[35] Leggett HC , El Mouden C , Wild G , West S . 2012 Promiscuity and the evolution of cooperative breeding. Proc. R. Soc. B **279** , 1405–1411. (10.1098/rspb.2011.1627) PMC3282361 21993501

[36] Wild G , Koykka C . 2014 Inclusive-fitness logic of cooperative breeding with benefits of natal philopatry. Phil. Trans. R. Soc. B **369** , 20130361. (10.1098/rstb.2013.0361) 24686933 PMC3982663

[37] Taylor PD , Frank SA . 1996 How to make a kin selection model. J. Theor. Biol. **180** , 27–37. (10.1006/jtbi.1996.0075) 8763356

[38] Frank SA . 1998 Foundations of social evolution. Princeton, NJ: Princeton University Press.

[39] Rodrigues AMM , Gardner A . 2022 Reproductive value and the evolution of altruism. Trends Ecol. Evol. **37** , 346–358. (10.1016/j.tree.2021.11.007) 34949484

[40] Gardner A , West SA , Wild G . 2011 The genetical theory of kin selection. J. Evol. Biol. **24** , 1020–1043. (10.1111/j.1420-9101.2011.02236.x) 21371156

[41] Rodrigues AMM , Gardner A . 2023 Transmission of social status drives cooperation and offspring philopatry. Proc. R. Soc. B **290** , 20231314. (10.1098/rspb.2023.1314) PMC10685119 38018113

[42] Frank SA . 1985 Hierarchical selection theory and sex ratios. II. On applying the theory, and a test with fig wasps. Evolution **39** , 949–964. (10.1111/j.1558-5646.1985.tb00440.x) 28561492

[43] Frank SA . 1987 Variable sex ratio among colonies of ants. Behav. Ecol. Sociobiol. **20** , 195–201. (10.1007/bf00299733)

[44] Yamaguchi Y . 1985 Sex ratios of an aphid subject to local mate competition with variable maternal condition. Nature **318** , 460–462. (10.1038/318460a0)

[45] Rodrigues AMM , Gardner A . 2016 The constant philopater hypothesis: a new life history invariant for dispersal evolution. J. Evol. Biol. **29** , 153–166. (10.1111/jeb.12771) 26431821 PMC4738439

[46] Johnstone RA . 2008 Kin selection, local competition, and reproductive skew. Evolution **62** , 2592–2599. (10.1111/j.1558-5646.2008.00480.x) 18691258

[47] Platt TG , Bever JD . 2009 Kin competition and the evolution of cooperation. Trends Ecol. Evol. **24** , 370–377. (10.1016/j.tree.2009.02.009) 19409651 PMC5679087

[48] Lehmann L , Rousset F . 2010 How life history and demography promote or inhibit the evolution of helping behaviours. Phil. Trans. R. Soc. B **365** , 2599–2617. (10.1098/rstb.2010.0138) 20679105 PMC2936172

[49] Kennedy P , Higginson AD , Radford AN , Sumner S . 2018 Altruism in a volatile world. Nature **555** , 359–362. (10.1038/nature25965) 29513655 PMC5986084

[50] Perrin N , Mazalov V . 2000 Local competition, inbreeding, and the evolution of sex‐biased dispersal. Am. Nat. **155** , 116–127. (10.1086/303296) 10657181

[51] Rodrigues AMM , Johnstone RA . 2014 Evolution of positive and negative density-dependent dispersal. Proc. R. Soc. B **281** , 20141226. (10.1098/rspb.2014.1226) PMC4132684 25100700

[52] Ronce O , Promislow D . 2010 Kin competition, natal dispersal and the moulding of senescence by natural selection. Proc. R. Soc. B **277** , 3659–3667. (10.1098/rspb.2010.1095) PMC2982254 20591867

[53] Kreider JJ , Kramer BH , Komdeur J , Pen I . 2022 The evolution of ageing in cooperative breeders. Evol. Lett. **6** , 450–459. (10.1002/evl3.307) 36579168 PMC9783459

[54] Hill K , Hurtado AM . 1991 The evolution of premature reproductive senescence and menopause in human females. Hum. Nat. **2** , 313–350. (10.1007/bf02692196) 24222339

[55] Nattrass S *et al* . 2019 Postreproductive killer whale grandmothers improve the survival of their grandoffspring. Proc. Natl Acad. Sci. USA **116** , 26669–26673. (10.1073/pnas.1903844116) 31818941 PMC6936675

[56] Cant MA , Johnstone RA . 2008 Reproductive conflict and the separation of reproductive generations in humans. Proc. Natl Acad. Sci. USA **105** , 5332–5336. (10.1073/pnas.0711911105) 18378891 PMC2291103

[57] Johnstone RA , Cant MA . 2010 The evolution of menopause in cetaceans and humans: the role of demography. Proc. R. Soc. B **277** , 3765–3771. (10.1098/rspb.2010.0988) PMC2992708 20591868

[58] Ekman J , Griesser M . 2016 Siberian jays: Delayed dispersal in the absence of cooperative breeding. In Cooperative breeding in vertebrates (eds WD Koenig , JL Dickinson ), pp. 6–18. Cambridge, UK: Cambridge University Press. (10.1017/cbo9781107338357.002)

[59] Fuirst M , Strickland D , Freeman NE , Sutton AO , Ryan Norris D . 2023 Early-life sibling conflict in Canada jays has lifetime fitness consequences. Proc. R. Soc. B **290** , 20221863. (10.1098/rspb.2022.1863) PMC10113022 37072037

[60] Drobniak SM , Wagner G , Mourocq E , Griesser M . 2015 Family living: an overlooked but pivotal social system to understand the evolution of cooperative breeding. Behav. Ecol. **26** , 805–811. (10.1093/beheco/arv015)

[61] Griesser M , Drobniak SM , Nakagawa S , Botero CA . 2017 Family living sets the stage for cooperative breeding and ecological resilience in birds. PLoS Biol. **15** , e2000483. (10.1371/journal.pbio.2000483) 28636615 PMC5479502

[62] Bruintjes R , Taborsky M . 2011 Size-dependent task specialization in a cooperative cichlid in response to experimental variation of demand. Anim. Behav. **81** , 387–394. (10.1016/j.anbehav.2010.10.004)

[63] Smith MG , LaPergola JB , Riehl C . 2024 Workload inequality increases with group size in a cooperatively breeding bird. Anim. Behav. **207** , 87–99. (10.1016/j.anbehav.2023.10.015)

[64] Smith MG , Riehl C . 2022 Workload distribution and division of labor in cooperative societies. Q. Rev. Biol. **97** , 183–210. (10.1086/721520)

[65] Rodrigues AMM , Riehl C . 2025 Supplementary material from: The evolution of cooperative breeding in family groups: when should parents tolerate unhelpful helpers? Figshare. (10.6084/m9.figshare.c.7676038) PMC11969389 40109113

